# An Efficient Low Cost Method for Gene Transfer to T Lymphocytes

**DOI:** 10.1371/journal.pone.0060298

**Published:** 2013-03-26

**Authors:** Leonardo Chicaybam, Andressa Laino Sodre, Bianca Azevedo Curzio, Martin Hernan Bonamino

**Affiliations:** 1 Programa de Carcinogênese Molecular, Coordenação de Pesquisa (CPQ), Instituto Nacional de Câncer (INCA), Rio de Janeiro, Brazil; 2 Instituto de Pesquisa Clínica Evandro Chagas (IPEC), Fundação Instituto Oswaldo Cruz (FIOCRUZ), Rio de Janeiro, Brazil; Northwestern University Feinberg School of Medicine, United States of America

## Abstract

Gene transfer to T lymphocytes has historically relied on retro and lentivirus, but recently transposon-based gene transfer is rising as a simpler and straight forward approach to achieve stable transgene expression. Transfer of expression cassettes to T lymphocytes remains challenging, being based mainly on commercial kits.

**Aims:**

We herein report a convenient and affordable method based on *in house* made buffers, generic cuvettes and utilization of the widely available Lonza nucleofector II device to promote efficient gene transfer to T lymphocytes.

**Results:**

This approach renders high transgene expression levels in primary human T lymphocytes (mean 45%, 41–59%), the hard to transfect murine T cells (mean 38%, 36–42% for C57/BL6 strain) and human Jurkat T cell line. Cell viability levels after electroporation allowed further manipulations such as *in vitro* expansion and Chimeric Antigen Receptor (CAR) mediated gain of function for target cell lysis.

**Conclusions:**

We describe here an efficient general protocol for electroporation based modification of T lymphocytes. By opening access to this protocol, we expect that efficient gene transfer to T lymphocytes, for transient or stable expression, may be achieved by an increased number of laboratories at lower and affordable costs.

## Introduction

The genetic modification of T lymphocytes is a usual approach to study the biology of these cells and recently it has been largely used to generate tumor-specific effector cells. However, T cells have been proven difficult to modify using non-viral methods such as lipid-based plasmid transfection reagents, like Lipofectamine. These methods have been associated with high toxicity and low efficiency[1]. The development of retroviral/lentiviral vector based transduction led to efficient gene transfer when using T lymphocytes [2]–[6] and other hematopoietic cells [5], [7], being the method of choice for clinical trials using transgene-modified T cells. [6], [8]–[11]. However, such cell modification protocols are time-consuming and expensive, limiting a broader application in the clinical setting[12].

Electroporation has emerged as a powerful tool for the genetic modification of diverse cell types [13]–[15]. This technique is based on the transient disruption of cell membrane after exposure to an electric field, allowing charged molecules to enter the cell. The main potential disadvantages of this methodology are the excessive cell death and the low transfection efficiency, although these phenomena depend on the cell type used and electroporation conditions (including voltage and wave profile, time of the pulse and buffer composition). Indeed, early attempts of electroporating T cells with plasmid DNA resulted in low transgene expression [16], [17]. Several reports showed a high efficiency when using RNA [18], but this prevents the selection of cells with stable expression of the transgene. Of particular interest are the square-wave pulse based new electroporation devices, such as the broadly used Lonza Nucleofector II electroporation system, which showed high efficiency in the genetic modification of T cells applying proprietary electroporation buffers and electric parameters[19]. Using this methodology, according to Nucleofactor manufacturer, up to 80% of viability and 40–60% of expression was achieved in human T cells; in murine T cells, usually harder to transfect, 35–55% of viability and 20–40% of expression can be achieved, varying according to the mouse strain used.

The use of this platform in combination with a non-viral genetic modification system capable of inducing stable expression of the transgene, such as the Sleeping Beauty (SB) transposon, configures an interesting approach for the genetic modification of T cells. The SB system consists of a transposase that recognizes flanking repetitive sequences in the transposon plasmid containing the transgene and mediates its integration in the host genome [20]. It has emerged as a gene transfer system with potential to be broadly used in gene transfer and therapy protocols, accomplishing clinical grade requirements [21], with high efficiency in T cells. Due to a random integration profile, it minimizes the chance of insertional mutagenesis when compared with other transposon systems [22].

The adoptive transfer of tumor-specific lymphocytes is a promising new strategy for cancer treatment [23], [24]. However, this approach is largely dependent on tumor infiltrating lymphocyte expansion and/or T cell receptor (TCR) gene transfer [25]. More recently, T lymphocytes carrying Chimeric Antigen Receptor (CAR) molecules proved successful in clinical trials [9], [10], [26]. These receptors consist of an extracellular domain derived from an antibody that recognizes the antigen, a transmembrane domain and an intracellular signaling domain capable of activating the lymphocyte. Such a receptor allows the recognition of the antigen in a HLA-independent fashion. Despite the poor response observed in early clinical trials, which used CARs containing only the CD3zeta signaling domain, the addition of costimulatory motifs such as CD28 or 4-1BB augmented the function and persistence of these cells *in vivo*, achieving objective clinical responses in several patients (reviewed in [27]). Importantly, it was recently showed that the transferred cells can persist for more than a decade after the infusion [28]. Recent reports showed efficient generation of CAR-expressing T cells using the SB system [29]–[31], and this approach is already being used in clinical trials [21].

Despite remarkable gene transfer rates[19], the high cost of Lonza compatible electroporation kits and the dependence on manufacturer reagents and devices may limit the application of this technology for large scale experiments, especially in the research settings where multiple electroporations may be required, for instance when testing different CAR constructs or modifying gene expression in T lymphocytes targeting several genes. We herein describe a new set of generic buffers optimized for the genetic modification of human and murine T lymphocytes using Lonzàs Nucleofactor II device. The combined use of our buffers with the SB system allowed the generation of transgene expressing lymphocytes with high efficiency avoiding using commercial Lonza kits. This approach allows the extensive testing for best electroporation conditions and robust transient or stable gene expression for studies requiring gene transfer to T lymphocytes either for basic research or experimental immunotherapy protocols.

## Materials and Methods

### Donors

The use of peripheral blood mononuclear cells (PBMC's) from healthy donors was approved by an IRB (Brazilian National Cancer Institute - INCa - Ethics Committee) and donors signed review board approved informed consents. Within 24 h after blood collection, leukocytes were harvested by filtration and washed with Phosphate Buffered Saline (PBS) 1x. A density gradient centrifugation using Ficoll-Hypaque®-1077 was performed. Cells were centrifuged for 20 min at 890 g (slow acceleration/deceleration off), washed three times with PBS and used for transfection.

### Cell culture

Jurkat cell line (clone E6–1) was kindly provided by Dr. João Viola (INCa, Brazil). PBMC's were obtained from healthy donors, as described above. Mouse T lymphocytes were obtained from total lymph node (LN) removal (except mesenteric LN) of C57BL/6, BALB/c or B10A mice provided by INCa's animal facility. This study was carried out in strict accordance with the recommendations in the Guide for the Care and Use of Laboratory Animals of the Instituto Nacional de Cancer (Brazil). The protocol was approved by the Committee on the Ethics of Animal Experiments of the Instituto Nacional de Cancer (Brazil).

Human and mouse leukocytes were seeded in sterile 12-well plates and maintained in growth media. EBV-transformed B lymphoblastic cell line LAS 388 (L388) was provided by Dr. Alexandre Nowill (Unicamp, SP, Brazil) and expanded in sterile 75 cm2 flasks. Human pre-B NALM-6 cell line was a kind gift of Dr. Andrea Biondi (Fondazione M Tettamanti, Monza, Italy) [32]. All cells were grown in RPMI medium (Sigma, cat. R5886) supplemented with 10% Fetal Calf Serum (FCS - Cultilab, Brazil), 2 mM L-Glu (Gibco cat. 25030-081), 2-Mercaptoethanol (55 µM, Gibco, cat. 21985-023) and Penicillin/Streptomycin (Gibco, cat. 15070-063) at 37°C and 5% CO2. For primary lymphocyte culture (murine and human), 50 UI/mL of human rIL-2 (Aldesleukin) was added to the medium.

### Plasmids and Cloning

Transposon pT2-GFP and transposase SB100X [30] were kindly provided by Dr. Sang Wang Han (UNIFESP, Brazil). Transposon pT3-GFP [31] was provided by Dr. Richard Morgan (NIH, USA). The first generation CAR 20z, containing a CD20 antigen binding domain, a CD8α hinge and a CD3ζ signaling domains, was synthesized and codon-optimized by Genscript (NJ, USA) based on the sequence provided originally by Dr. Dario Campana (St. Jude Children's Research Hospital, Memphis, USA) for 20BBz CAR [33]. The sequence was linearized by AgeI and NotI digestion and inserted into pT3-GFP transposon backbone after GFP removal by the same enzymes.

### T cell activation and expansion

After transfection with pT2-GFP, human T cells were activated by 1 µg/mL OKT-3 (eBioscience, cat.: 16-0037) and 0.5 µg/mL aCD28 (eBioscience, cat.: 16-0289). Alternatively, expansion of CAR-transfected human T lymphocytes was performed by stimulation with γ-irradiated (100Gy) L388 cells at a ratio of 1∶5 (L388:T lymphocytes) and addition of 50 U/mL of human recombinant IL-2. Experiments using mouse cells were approved by our local (INCA) animal care and use committee. Mouse T cells were activated 24 h before electroporation using 2 µg/mL aCD3 (clone 2C11; purified in house) and 1 µg/mL aCD28 (clone 37.51, eBioscience, cat.: 16-0281) in the presence of 50 U/mL IL-2. After leukocyte transfection and stimulation, media was replaced every other day. Cells were expanded up to 12 days until all the analyses have been performed.

### Primary T cell electroporation

Non-viral transfection of human cells was performed using unstimulated PBMC from healthy donors. Mouse T lymphocytes were activated before electroporation as described above. A total of 10^7^ PBMC or mouse T lymphocytes were pelleted (200 g, 10 min) and resuspended in 100 µL pre-warmed electroporation buffer. The buffers used were provided from Lonza® Human T Cells Nucleofector® Kit for human T cells (cat number VPA-1002), Mouse T Cell Nucleofector® Kit for mouse T cells (cat number VPA-1006), or in house prepared buffer solutions for human and mouse lymphocytes (described in Table S1). For all the electroporations using in house buffers, generic cuvettes were used (Mirus Biotech®, Madison, WI, USA cat.: MIR 50121). Plasmid masses varying from 0 to 20 µg of the reporter gene or 20 µg of the 20z CAR encoding plasmid DNA (pT2-GFP or pT3-20z transposons, respectively) and 0 to 2 µg of SB100X transposase were added. The cells were immediately transferred to a sterile 0.2 cm cuvette and electroporated using the program U-14 for human T lymphocytes and X-001 for mouse T lymphocytes of Lonza® Nucleofector® II electroporation system. After transfection, mouse lymphocytes electroporated with Lonzás kit were gently resuspended in 1 mL of pre-warmed media supplied by the manufacturer and supplemented as described by the Lonza® kit manual. Mouse T cells transfected using in house solutions and all human T cells were gently resuspended in 1 mL of pre-warmed RPMI medium supplemented only with 2 mM L-Glu and 20% FCS. All cells were seeded in 12-well plates and grown overnight at 37°C and 5% CO2. The media was replaced by complete RPMI medium the following day and cells were maintained as described previously. The "electroporation score" was calculated based on cell viability (based on trypan blue exclusion and/or determination of the % of cells displaying viable cell FSC vs SSC parameters by flow cytometry analysis) and transgene expression, and the score set to the formula "Viability (%)*Expression (%)/50". A division factor was used in the score to fit the results in the graph scale.

### Jurkat electroporation

Prior to electroporation, 1 ×10^6^ Jurkat cells were centrifuged at 90 g for 10 minutes, resuspended in 100 µL of the desired electroporation buffer and mixed with 4 µg of pT2-GFP plasmid.

The resuspended cells were transferred to cuvettes and immediately electroporated using the program X-005. After electroporation, cells were incubated in the cuvette at room temperature for ten minutes and then 500 µL of pre-warmed RPMI medium supplemented with 20% FCS were added to the cuvette. Cells were transferred to a 12-well plate and incubated at 37°C5%CO2 overnight. The day after electroporation, cells were centrifuged and medium was replaced by RPMI supplemented with 10% FCS.

Cell viability and percentage of GFP+ cells were evaluated by flow cytometry on days 1 and 2 after electroporation by excluding cells from previously determined viable cells gate. The "electroporation score" was calculated based on cell viability and transgene expression values, and the score set to the formula "Viability (%)*Expression (%)/20". A division factor was used in the score to fit the results in the graph scale.

### Flow cytometry

FACSCalibur® (BD Bioscience) was used to perform morphologic evaluation of viability (FSC vs SSC) and transgene expression analysis. Cells were harvested the following days after transfection and resuspended in PBS 1x at a concentration of 10e5 cells/500 µL. CAR-transfected T lymphocytes were stained using biotin-conjugated anti Fab goat mAb, (Jackson ImmunoResearch, cat. 115-065072) for 20 min at 4°C and then washed with PBS 1x before staining with streptavidin-PECy-5 (eBioscience; cat. 15431782) for flow cytometry analysis. CD20 staining was performed with anti-CD20 APC (eBioscience, cat. 17020942). 7AAD staining (eBioscience cat. 00-6693) was performed immediately before FACS acquisition following manufacturer instructions.

### *In vitro* cytotoxicity of T lymphocytes

Cytotoxic T lymphocytes were incubated with CD20+ Nalm-6 GFP+ target cells (90% GFP+/85% CD20+; 10^5^ per well) at different effector to target (E/T) ratios (6∶1; 3∶1, 1,5∶1; 0,75∶1; 0,375∶1 and 0,18∶1) in 96-well plates. After 4 hours at 37°C, the cells were harvested and the percentage of CD20+/GFP+ cells was analyzed by flow cytometry. The percentage of specific lysis was calculated dividing the %GFP+ cells observed by the % of GFP cells expected in each E/T ratio.

### Statistical analysis

**Figure 1:**
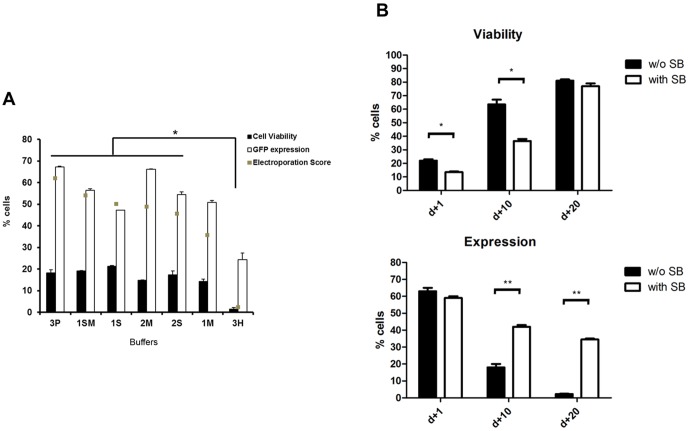
Electroporation of the Jurkat T cell line. (**A**) Jurkat cells were electroporated with pT2-GFP in the presence of each of the 7 different buffers. Viability and GFP expression were evaluated by flow cytometry after 24 h. Cell viability is expressed as % of the control mock electroporated condition (100%). Electroporation scores were determined as described in materials and methods. Statistical analysis was performed using One Way ANOVA and Tukey post test (* = P<0.05). (**B**) Jurkat cells were electroporated with buffer 3P. Cell viability and GFP expression were observed until day 20. Values in this figure are the average of two separate experiments in triplicate and are expressed as mean±SEM. Data were analyzed by unpaired Student t test; p<0.05 (*);p<0.01 (**).

Results are shown as means±s.e.m. Comparisons between Lonzás kit and in house buffers and conditions with or without SB (Fig 1B) were performed using unpaired Students *t* test. P<0.05 was considered statistically significant.

For figures comparing electroporation scores, statistical analysis was performed using One Way ANOVA and Tukey post test (P<0.05).

## Results

### Jurkat cell line electroporation

To test our in house buffers (described in Table S1) in the genetic modification of a relevant cell line, we electroporated the cell line Jurkat, a model of T lymphocytes, with a transposon transfer cassette carrying GFP. Cell viability was based on viable FSC/SSC parameters gate throughout the manuscript since cells gated this way proved to be 7AAD negative in primary T lymphocytes (Figure S1). The percentage of GFP+ cells were evaluated by flow cytometry. We observed no statistically significant differences among cells electroporated with buffers 1M, 1SM, 1S, 2M, 2S and 3P for day +1 (Figure 1A). The program X-005 was used, as it resulted in better scores than program X-001 (data not shown). The highest electroporation score was obtained for 3P buffer. After 20 days in culture, Jurkat cell viability reached levels superior to 80% as evaluated by trypan blue exclusion (data not shown) and flow cytometry (Figure 1B), indicating that cell culture viability can recover after the electroporation protocol. Long term expression for buffer 3P was maintained, with 35% of cells expressing GFP on d+20 (Figure 1B).

### High electroporation efficiency of primary human T cells can be achieved using in house solutions

**Figure 2:**
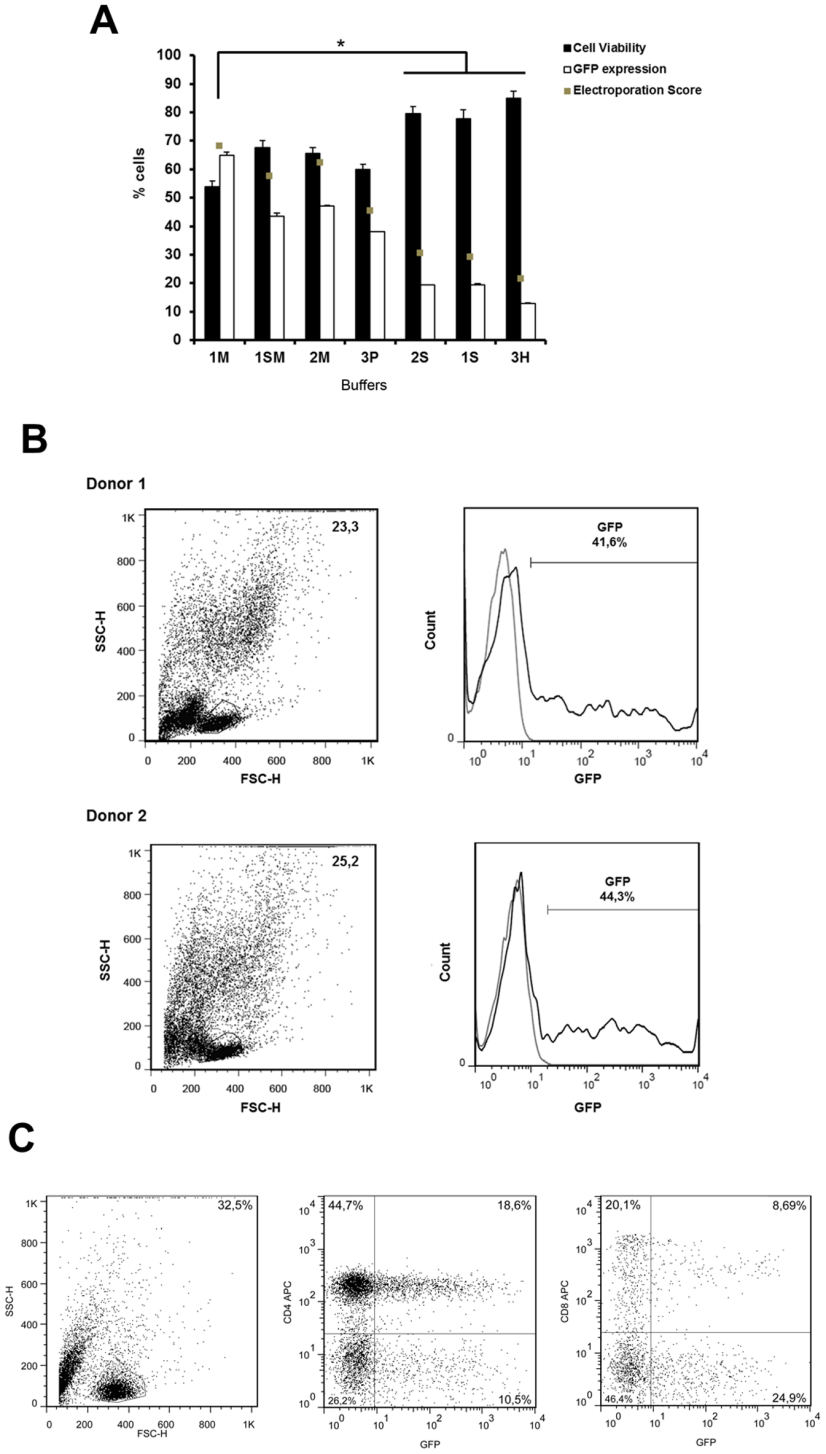
Electroporation efficiency of different buffers in primary human T lymphocytes. (**A**) PBMCs from two healthy donors were electroporated using in house buffers and 4 µg of pT2-GFP plasmid. Cell viability and GFP expression were analyzed after 24h by flow cytometry. Cell viability is normalized to control (not electroporated) cells. Electroporation scores were determined as described in materials and methods. Values are the average of two donors in triplicate and are expressed as mean±SEM. Statistical analysis was performed using One Way ANOVA and Tukey post test (* = P<0.05). (**B**) Representative dot plots of GFP (pT2-GFP; 4 µg) expression 24 h after electroporation with 1SM buffer. Numbers in plots represents the percentage of cells in the gate. Gray line = negative control; black line = cells electroporated with pT2-GFP. (**C**) Electroplated lymphocytes were stained for CD4 and CD8 24 h after electroporation with 1SM buffer and 4 µg pT2-GFP plasmid. Data are representative of two donors. Numbers in plots represents the percentage of cells in the gate.

**Figure 3:**
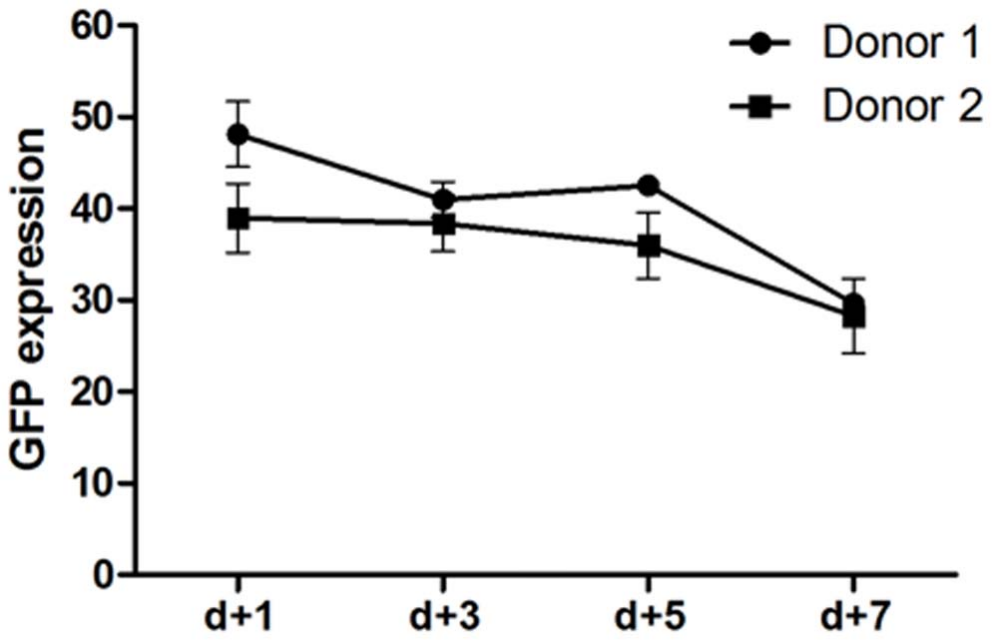
Long term expression of GFP after electroporation with buffer 1SM. PBMCs from two healthy donors were electroporated using 1SM buffer and 4 µg of pT2-GFP plasmid. After 24 h cells were activated with anti-CD3/anti-CD28 and GFP expression was evaluated for 7 days by flow cytometry.

We tested our in house transfection buffers in primary human T cells by GFP transfection of fresh PBMC from two healthy donors. To determine electroporation efficiency, cell viability and GFP expression were evaluated after transfection using 0.5 µg of SB100X transposase [30] and 4 µg of pT2-GFP transposon (plasmid mass recommended by Lonza). Figure 2A demonstrates that buffers 1M, 1SM, 2M and 3P maintained the viability of the cells above 50% and achieved superior expression of GFP at day 1 after transfection (representative plots are shown in Figure 2B); accordingly, high rates of expression were sustained up to 7 days with several buffers (Figure S2). As recommended by Lonza for unstimulated T cells, the program U-14 was used with better results than program V-024 (data not shown). Equivalent levels of transgene expression could be observed in CD4+ and CD8+ T lymphocytes after electroporation, while NK (CD56+) cells did not display transgene expression (data not shown and Figure 2C). Electroporation of lymphocytes with PT2-GFP (solution 1SM) followed by activation with anti-CD3 and anti-CD28 antibodies proved that the expression of the transgene could be retained at levels around 30% until d+7 (Figure 3). Since T lymphocyte culture is known as highly sensitive to cell death, diminished viability after electroporation may impact the maintenance of the culture in the long term. Because electroporation results with solution 1SM showed slightly better viability until day 3 (Figure S2), which could be crucial for recovery of viable human T lymphocytes, this buffer was used in our following tests despite the highest electroporation scores obtained with buffers 1M and 2M.

### Impact of plasmid mass used in electroporation on cell viability and transgene expression

**Figure 4:**
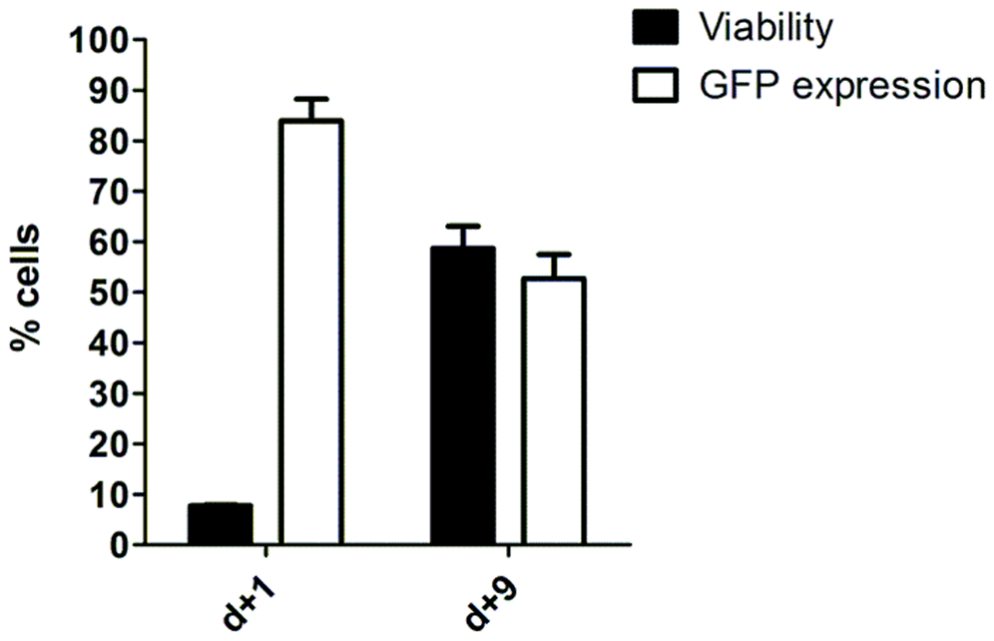
Electroporation of transposon and transposase maintains transgene expression after cell viability recovery. PBMCs from two healthy donors were electroporated using 1SM buffer, 20 µg of pT2-GFP plasmid and 2 µg of SB100x transposase. Cell viability and GFP expression were analyzed by flow cytometry on day 1 and 9. Values are the average of two donors in triplicate and are expressed as mean±SEM.

To evaluate the impact of different amounts of transposase in GFP expression, transgene integration and cell viability, we determined the best ratio of transposon/transposase for electroporation of fresh PBMCs from two healthy donors. Figure 4 shows that 2 µg of SB100X transposase maintained a high percentage of GFP (>50%) at the end of 9 days despite a loss (nearly 30%) of expression since day 1 after electroporation. Results obtained with these donors had lower initial viability probably due to the toxicity of higher mass of plasmid used (20 µg in Figure 4 vs 4 µg in Figure 2, also observed in Figure S3) and the percentage of viable cells increased at day 9. Increasing the amount of transposase up to 2 µg is paramount to sustain transgene expression showing almost no impact in cell viability while the absence of transposase resulted in reduced GFP percentages on later days (Figure S3), indicating the crucial role of the enzyme in maintaining transgene expression. Increasing amounts of pT2-GFP plasmid also impacted the percentage of GFP positive cells and the levels of GFP fluorescence as indicated by higher mean fluorescence intensity according to pT2-GFP mass increase from 4 to 20 µg. When 20 µg of plasmid mass is used with 0.5, 1 or 2 µg of SB100X, cell viability may be decreased until day 5 after electroporation (Figure S3).

**Figure 5:**
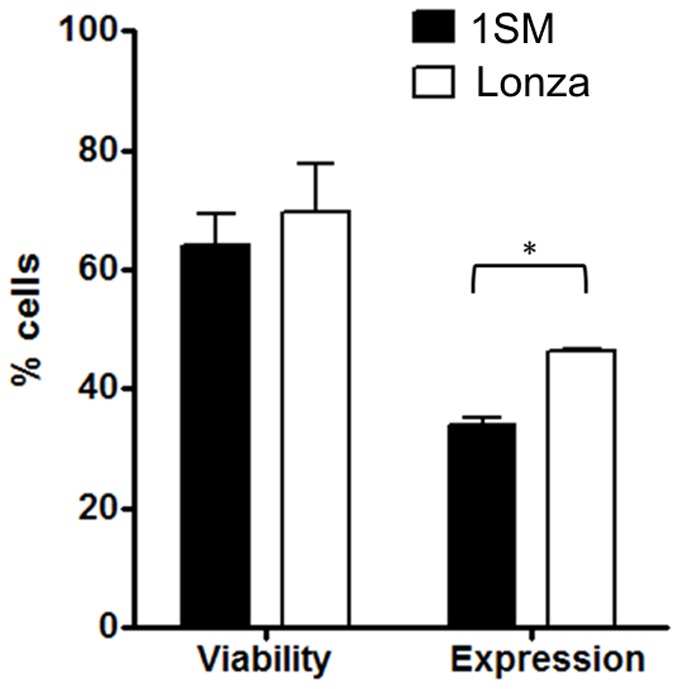
Comparison of in house vs commercial electroporation kit. PBMCs from two healthy donors were electroporated using either 1SM buffer or Lonza buffer and 4 µg of pT2-GFP plasmid. Cell viability and GFP expression were analyzed after 24 h by flow cytometry. Cell viability was normalized to control (not electroporated) cells. Values are the average of two donors in triplicate and are expressed as mean±SEM. Data were analyzed by unpaired Student t test; p<0,05 (*).

When our electroporation buffer 1SM was compared to the commercially available Lonza® Kit specific for human T lymphocytes, it showed no significant difference in cell viability, but the levels of GFP expression were slightly lower. The electroporation scores for 1SM buffer and Lonza kit were 43,5 and 64,4 respectively (Figure 5).

### Mouse T cells can be electroporated using in house buffers

**Figure 6:**
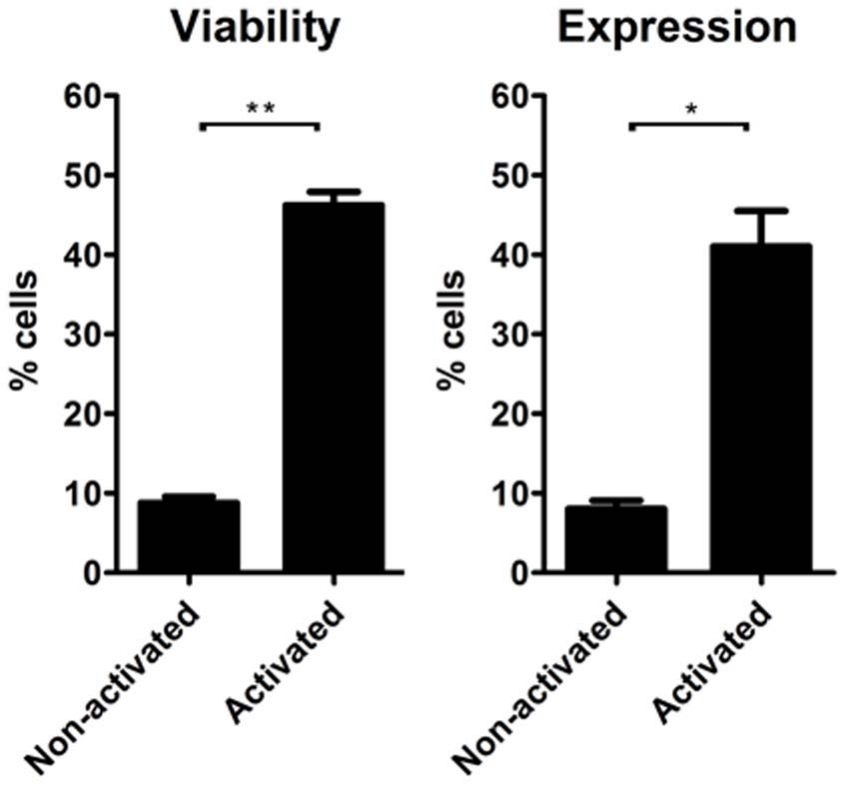
Electroporation of resting vs activated mouse primary T lymphocytes. Total lymphocytes from lymph nodes of C57BL/6 mice were isolated and either directly electroporated or activated for 24 h with anti-CD3/anti-CD28 and then electroporated. Buffer 2S and 4 µg of pT2-GFP plasmid were used. Cell viability and GFP expression were analyzed after 24 h by flow cytometry. Cell viability is normalized to control (not electroporated) cells. Data are representative of two experiments in triplicate and expressed as mean±SEM. Data were analyzed by unpaired Student t test; p<0.0001 (**) or p = 0.002 (*).

To determine the possibility of electroporating murine T lymphocytes using our home made solutions, we first determined the impact of the activation status of T lymphocytes on the survival to the electroporation process. Pre activating lymph node isolated lymphocytes with anti CD3/CD28 antibodies led to remarkably better survival of cells of C57BL/6 mouse strain after electroporation (Figure 6). All activated lymphocytes reached superior cell viability than the non-activated populations (more than 30% compared to less than 10%), and GFP transfection was approximately four times higher among cells activated before electroporation. These data demonstrates that pre-activation is mandatory to obtain viable electroporated mouse T cells in our system.

**Figure 7:**
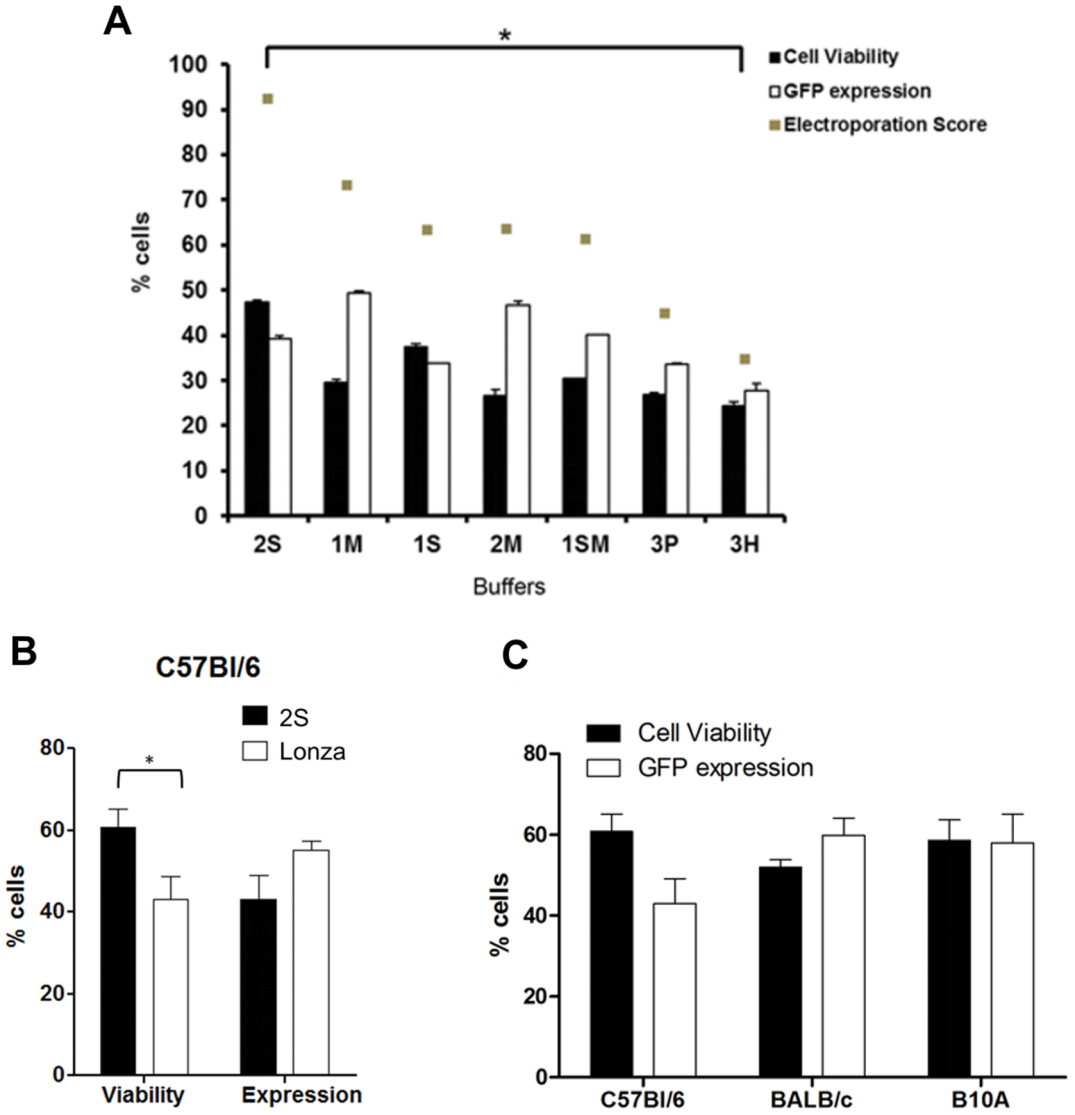
Efficiency of murine T lymphocyte electroporation is dependent on different buffer and mouse strain. (**A**) Total lymphocytes from lymph nodes of C57BL/6 mice were isolated and electroporated using in house buffers and 4 µg of pT2-GFP plasmid. Cell viability and GFP expression were analyzed after 24 h by flow cytometry. Electroporation scores were determined as described in materials and methods. Statistical analysis was performed using One Way ANOVA and Tukey post test. P<0.05 (*). (**B**) Total lymphocytes from lymph nodes of C57BL/6 mice were isolated and electroporated using 2S buffer or Lonza buffer and 4 µg of pT2-GFP. Analysis was performed 24 h later by flow cytometry. Data were analyzed by unpaired Student t test p<0,05 (*). (**C**) Total lymphocytes from lymph nodes of C57BL/6, Balb/c and B10A mice were isolated and electroporated using 2S buffer and 4 µg of pT2-GFP plasmid. Cell viability was normalized to control (not electroporated) cells. All values in this figure represent the average of two experiments in triplicate and are expressed as mean±SEM.

We next tested our different buffers to determine the most appropriated for mouse T cells. Lymphocytes obtained from lymph nodes of C57BL/6 mice were electroporated using the buffers described in Table S1. Comparison between solutions demonstrated that higher GFP expression was achieved after electroporation with 1M and 2S buffers, with percentages of positive cells greater than 35% (Figure 7A). Since cell viability and the final electroporation score showed superior when using solution 2S, this buffer was selected to perform the following assays.

To compare the results of solution 2S with Lonza's kit, mouse lymphocytes from lymph nodes (C57BL/6 strain) were isolated, electroporated following Lonza's kit instruction using the recommended nucleofector II program and compared to results obtained using 2S solution. As demonstrated in Figure 7B, our buffer achieved superior cell viability while maintaining similar GFP expression and a slightly higher electroporation score (52,8) than the Lonza group (47,4). Since the manufacturer of the reference commercial electroporation kit indicates that different mouse strains can show wide variation in electroporation efficiencies, we tested the electroporation of 3 different mouse strain lymphocytes (C57BL/6, BALB/c and B10A) side by side. We observed no substantial variation when different mouse strains were compared using solution 2S (Figure 7C); as in C57BL/6, buffers 1M, 1S and 3P achieved a lower efficiency when compared to buffer 2S (data not shown). It is important to note that we were unable to achieve long term expression of the transgene when SB transposase was used under the conditions tested (data not shown).

Since Flanagan and colleagues have shown that certain polymers can boost the transfection efficiency for some cells [34], we asked whether different amounts of polyethylene glycol (PEG - 0.01, 0.1 and 1%) added to 2S buffer during electroporation could augment the number of viable cells and transgene expression in mouse T cells. We observer no positive impact on cell viability whereas slight variations in GFP expression were observed; however, solution 2S achieved higher percentages of GFP positive cells in the absence of PEG (Figure S4). As in the control (solution 2S) condition, pre activation of T cells had deep impact in cell viability. Other polymer tested (Poloxamer-188) showed no significant impact in electroporation efficiency at 0.01, 0.1 or 1% concentrations and further appeared to slightly decrease the percentage of GFP-positive lymphocytes in the non-activated population, showing no effect on transfection of activated T cells (Figure S5).

### Efficient CAR-transfection of human T cells

**Figure 8:**
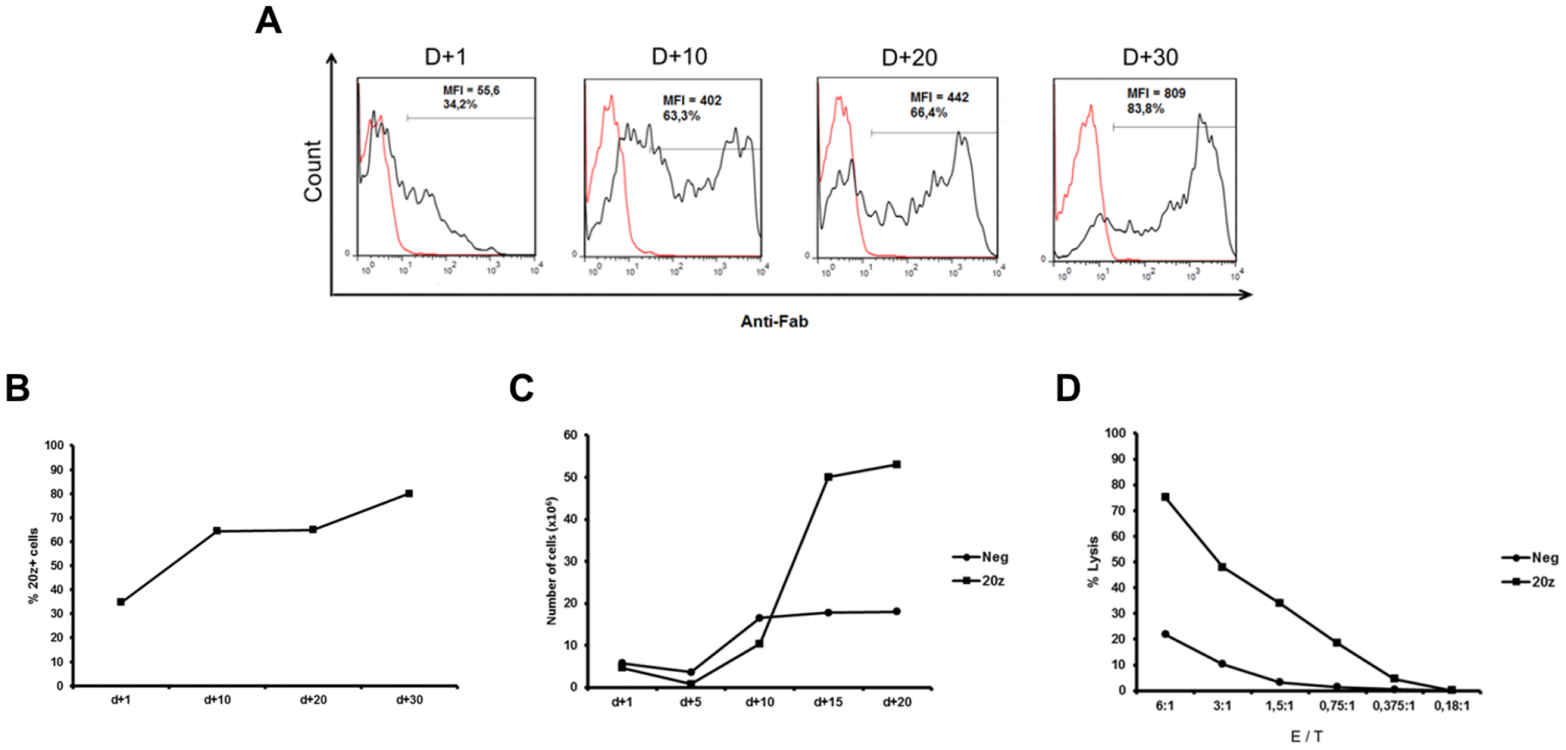
T cell electroporation with Chimeric Antigen Receptor (CAR) results in stable gene expression conferring target-specific cytotoxicity. PBMCs from one donor were electroporated using 1SM buffer, 20 µg of pT3-20z plasmid and 0,5 µg of SB100x transposase plasmid. One day later, lymphocytes were stimulated with irradiated L388 cells and CAR expression was evaluated until d+30 by flow cytometry analysis using an anti-fab antibody. (A) Representative histograms of 20z CAR expression at d+1, d+10, d+20 and d+30 after electroporation (gray line = non electroporated cells stained anti-Fab; black line = lymphocytes electroporated with pT3-20z). (B) Analysis of 20z CAR expression until d+30 summarizing data of Figure 8A. (C) Kinetics of expansion of control mock electroporated (Neg) or 20z+ lymphocytes after stimulation with L388 cells. (D) Expanded cells were used in different effector/target (E/T) ratios in a cytotoxic assay against the CD19+/CD20+ Nalm-6 GFP+target cell line; Neg = CTLs not electroporated.

To test whether solution 1SM was able to efficiently transfect human cells with the clinically relevant CAR 20z, we transfected fresh PBMC from a healthy donor and evaluated the percentage of transgene expression at 24 µh and after the stimulation with irradiated EBV+ B lymphoblastic L388 cell line. Analysis at days +1, +10, +20 and +30 showed that the expression of CAR was 34.7%, 63.3, 66.4% and 83.8% respectively, with a progressive increase in the percentage and MFI of CAR+ cells (Figures 8A and 8B). At day 20 after CAR transfection, expansion of cytotoxic T lymphocytes (CTLs) led to an absolute number of lymphocytes exceeding 5×10^7^ cells, with 20z+ cells having a clear proliferative advantage over mock electroporated (Neg) cells (Figure 8C). As showed in Figure 8D, T lymphocytes expressing 20z CAR displayed a high cytotoxic activity against the engineered CD20+ NALM-6 cell line when compared to control cells expanded in the same way but expressing no CAR, showing the feasibility of this protocol for the generation of antitumor specific T lymphocytes.

## Discussion

Given the importance of genetic modification of T cells for the study of T cell biology and immunotherapy, efficient and affordable methods to transfer genes to T lymphocytes are extremely useful to advance in these fields [12]. Human and, notably, mouse T lymphocytes are usually hard to electroporate and the emergence of systems like Lonza Nucleofector significantly improved transfection results. When large number of electroporations must be performed, costs associated with the acquisition of the electroporation kits can potentially limit its broad application. We herein report the development of in house buffers for the nucleofection of T cells, comprising different compositions and osmolarities, which allowed optimization of protocols for each target cell to be genetically modified (the results are summarized in Table S2).

The Jurkat T cell line, a prototype CD4+ lymphocyte used in many cell signaling studies, could be efficiently electroporated with our protocol yielding high expression of the reporter gene but low viability. Although the initial level of cell viability precludes refined cell analysis in the first 72 hours, cells are able to recover, allowing long-term evaluation of gene expression when SB100x transposase was included. For human primary T cells, several buffers resulted in high electroporation scores, with expression in both CD4+ and CD8+ subpopulations (Figure 2C). Surprisingly, GFP expression was not detected in NK cells, despite the presence of this cell subset (data not shown), indicating that the main cell population receiving and expressing the transgene are T lymphocytes. NK cells were previously described as hard to transfect and electroporate, but some protocolos for exogenous gene expression in these cells – NK-92 cell line and primary NK cells - were described [35]–[38]. We have no explanation for the observed restriction of NK cells to electroporation under the conditions tested in this report and NK cell resistance is currently being addressed by our group.

Electroporation of activated human T cells was ineffective with all the buffers and programs tested resulting in large loss of cell viability due to electroporation (data not shown). These results suggest that, at least for the conditions we tested, activated T lymphocytes are extremely sensitive to the provided electric pulse and do not allow efficient genetic modification if activated. These results prompted us to explore resting T cell electroporation followed by activation.

For murine primary T cells, only anti-CD3 activated lymphocytes were effectively electroporated, with little difference between T cells from Balb/c, C57BL/6 and B10A mouse strains tested. These results suggest a complete different behavior and capacity to recover from the electric pulse for murine T cells if compared to their human counterparts. We compared our protocol to Lonza kits and found no major differences in electroporation efficiencies when testing both human and murine primary cells. These results establish the buffers described in our report as reliable alternatives to Lonza's kits. The attempt to increase electroporation efficacy by adding polymers (PEG and Poloxamer-188) to electroporation buffers showed no impact in the level of transgene expression or cell viability obtained. Testing of additional polymers may increase the efficiency of the protocol and is currently under evaluation.

The combined use of the in house buffers with the non-viral SB system consists in a valuable tool for stable genetic modification. Although in this report we did not unequivocally demonstrate the integration of transgenes in host cell DNA, long term expression of GFP (Figure 1B) and CAR (Figures 8A and B) support the stable integration and expression of the transgenes transferred under the conditions tested. For mature T cells the risk of insertional mutagenesis is still theoretical [39], [40] and the integration pattern of SB transposase in these cells seems fairly random, unlike *piggyBac* or Tol2 that have a γ retrovirus-like profile [22]. These particular aspects indicate that using SB based technology combined with electroporation protocols represents one of the safest ways to transfer new genes to T lymphocytes. One potential limitation of the SB system is the overproduction inhibition, causing low transposition rates at high transposase concentration [41]. Furthermore, when expressed at high levels, SB transposase exhibits a cytotoxic effect dependent of p53 and c-Jun [42], although we did not observe reductions in GFP expression due to this phenomenon despite the different proportions of pT2/SB tested (Figures 4 and S3) in primary human lymphocytes. The ideal proportion of pT2/SB must be empirically established for each plasmid and cell type.

Most of data reported so far describe the electroporation of RNA molecules in T lymphocytes [18], [43]–[47] with very few reports addressing functional gene transfer with plasmids carrying the transgene [48] The limited number of reports using plasmid electroporation into lymphocytes precludes consistent comparisons to the results reported here. The current conditions described for electroporation and cell expansion lead to an efficient long term maintenance of primary human T cells carrying and expressing functional transgenes. Singh and colleagues [48] recently described a similar nucleofection system based on SB carrying CARs. Amongst the results described here, levels of transgene expression at day one after electroporation, presence of transgene protein in both CD4 and CD8 cells and the expansion rate of T lymphocytes after electroporation were very similar to those described by Singh and colleagues, reinforcing the equivalence of our in house system and the commercial available kits.

Finally, the new buffers described in this report are extremely useful in the context of experimental adoptive immunotherapy, specifically for the generation of antitumoral lymphocytes. As showed in Figure 8(A-D), long term expression of 20z CAR was successfully achieved conferring high cytotoxic activity against the engineered CD20+ Nalm-6 cell line to gene modified lymphocytes. Ongoing clinical trials performed elsewhere using similar methodologies to generate SB-modified CAR+ T cells[19], [21] reinforce the applicability of our method. Thus, the method presented here provides the researcher with independence over kits manufacturer, allowing functional studies under relevant conditions at similar efficiencies if compared to commercial kits, expanding the possibility of evaluating T cell function even in large scale experiments requiring multiple electroporations of primary T cells. Furthermore, the approach described here represents a viable alternative to more labor-intensive and time consuming retro/lentiviral vector based gene transfer methods, which usually require 2-3 weeks for vector production, titration and quality control testing. Moreover, our formulation provides low-income laoboratories with an affordable protocol of nucleofection, making the genetic modification of T cells a straight forward and accessible method.

## Supporting Information

Figure S1 **Analysis of 7AAD staining after electroporation.** PBMCs from three healthy **(A, B and C)** donors were electroporated using 1SM buffer and 2 µg of pT2-GFP plasmid. After 24 h, cells were stained with 7AAD and the viability was analyzed in gated (living cells as defined by scatter) or non-gated populations.
(TIF)

Figure S2 **Electroporation efficiency of different buffers.** PBMCs from two healthy donors were electroporated using in house buffers and 4 µg of pT2-GFP plasmid. Cell viability and GFP expression were analyzed until d+7 by flow cytometry. Values are the average of triplicates and expressed as mean±SEM. Viability of electroporated cells were normalized to the negative control (not electroporated) cells.
(TIF)

Figure S3 **Impact of different transposon and transposase plasmid mass in viability and transgene expression.** PBMCs from two healthy donors were electroporated using 1SM buffer, 4 µg, 10 µg or 20 µg of pT2-GFP plasmid and/or 0,5 µg, 1 µg or 2 µg of SB100x transposase plasmid. Cell viability and GFP expression were analyzed until d+9 by flow cytometry. Values are the average of two donors in triplicate and expressed as mean±SEM. Viability of electroporated cells were normalized to the negative control (not electroporated) cells.
(TIF)

Figure S4 **Electroporation of mouse lymphocytes in the presence of PEG.** Total lymphocytes from lymph nodes of C57Bl/6 mice were isolated and electroporated using 2S buffers (supplemented or not with PEG) and 4 µg of pT2-GFP plasmid. Cell viability and GFP expression were analyzed after 2 h by flow cytometry. Data is representative of two independent experiments in triplicate.
(TIF)

Figure S5 **Electroporation of mouse lymphocytes in the presence of Poloxamer-188.** Total lymphocytes from lymph nodes of C57Bl/6 mice were isolated and electroporated using 2S buffers (supplemented or not with Poloxamer-188) and 4 µg of pT2-GFP plasmid. Cell viability and GFP expression were analyzed after 24 h by flow cytometry. Data is representative of two independent experiments in triplicate.
(TIF)

Table S1 Buffers used in electroporation experiments.
(DOCX)

Table S2 Summary of the results obtained with in house buffers in different cell types.
(DOC)
