# Supplementary figures and images for: An Efficient Low Cost Method for Gene Transfer to T Lymphocytes
Source: PLoS One. 2013 Mar 26;8(3):e60298. doi: 10.1371/journal.pone.0060298 (PMC3608570; doi:10.1371/journal.pone.0060298)

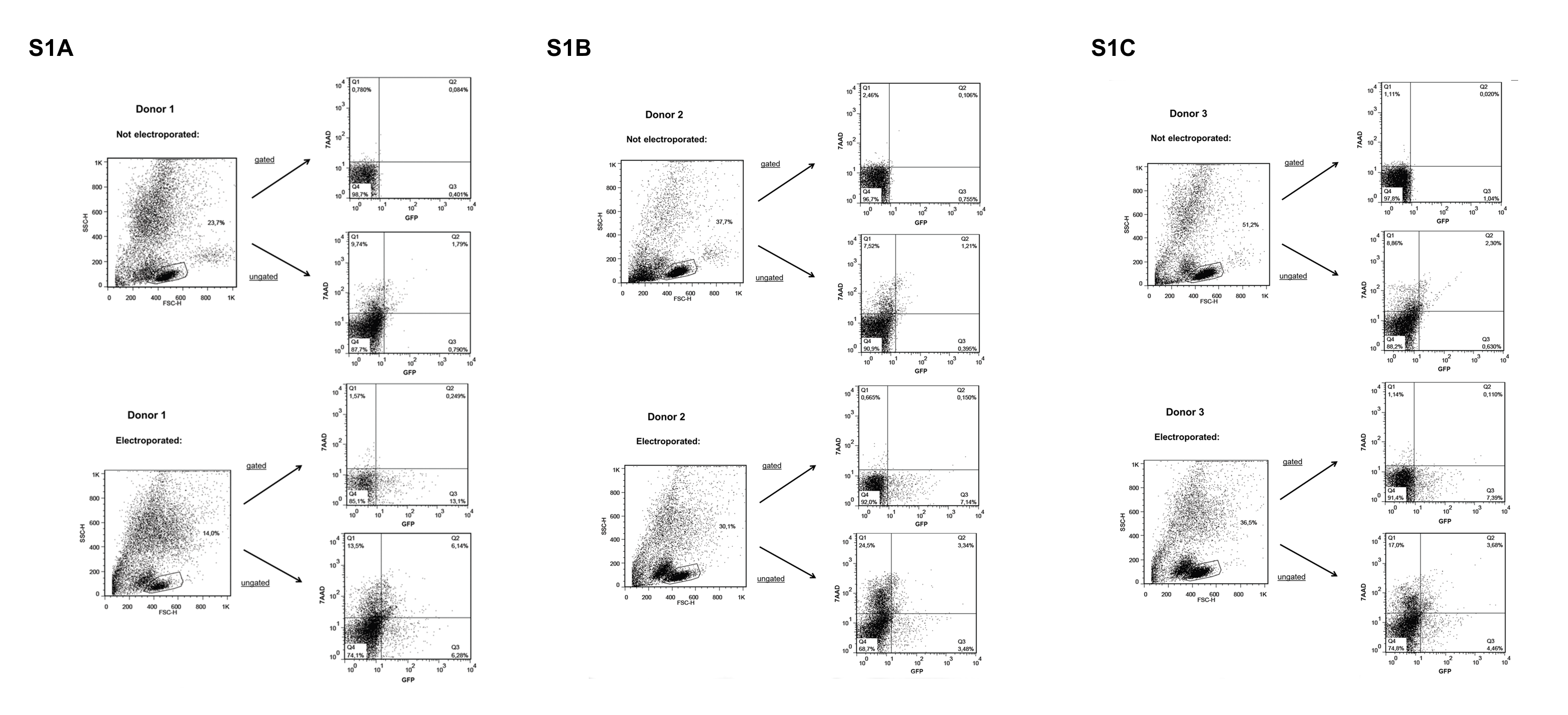

Supplement: Figure S1 — Analysis of 7AAD staining after electroporation. PBMCs from three healthy (A, B and C) donors were electroporated using 1SM buffer and 2 µg of pT2-GFP plasmid. After 24 h, cells were stained with 7AAD and the viability was analyzed in gated (living cells as defined by scatter) or non-gated populations. (TIF) [file pone.0060298.s001.tif]

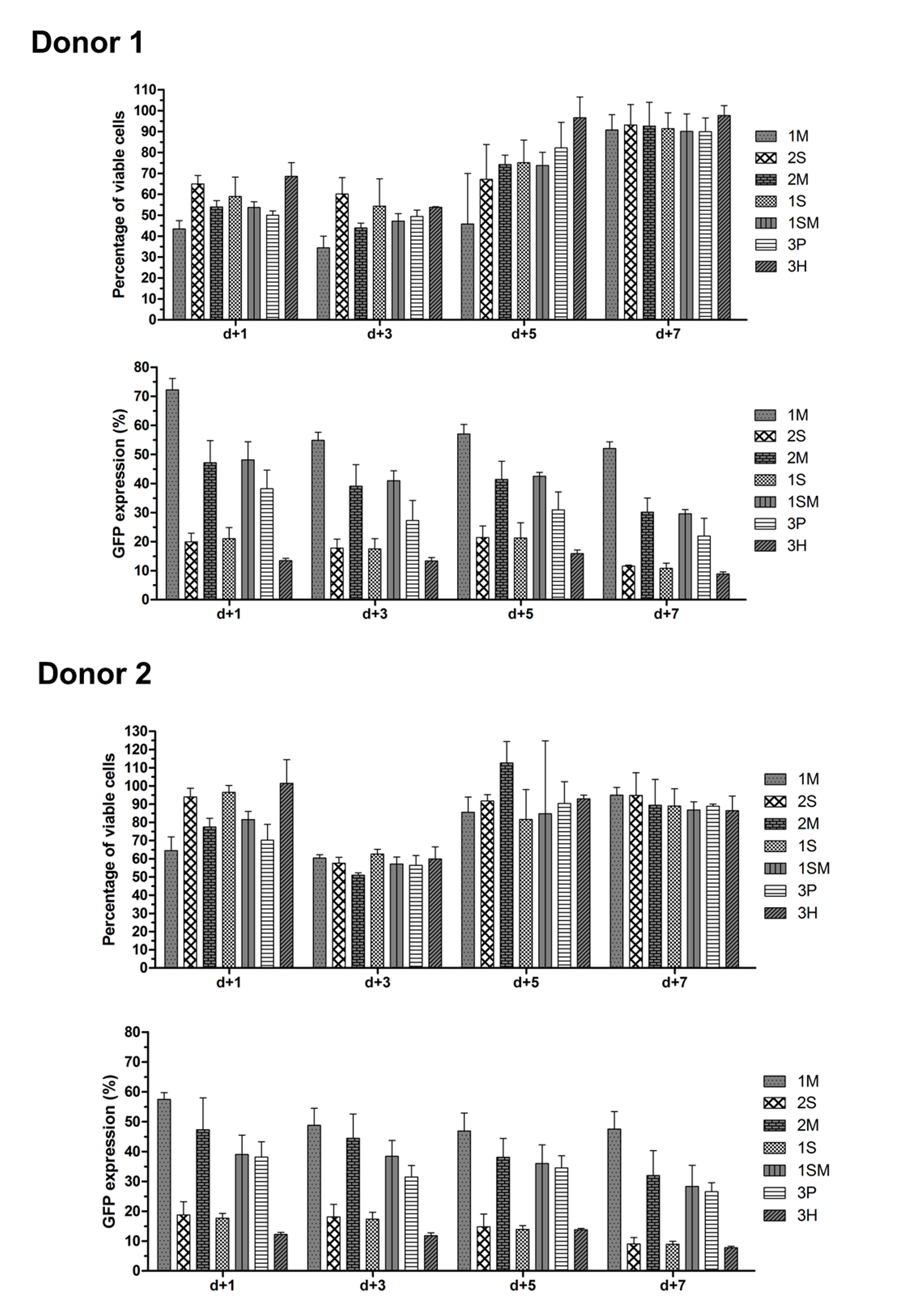

Supplement: Figure S2 — Electroporation efficiency of different buffers. PBMCs from two healthy donors were electroporated using in house buffers and 4 µg of pT2-GFP plasmid. Cell viability and GFP expression were analyzed until d+7 by flow cytometry. Values are the average of triplicates and expressed as mean±SEM. Viability of electroporated cells were normalized to the negative control (not electroporated) cells. (TIF) [file pone.0060298.s002.tif]

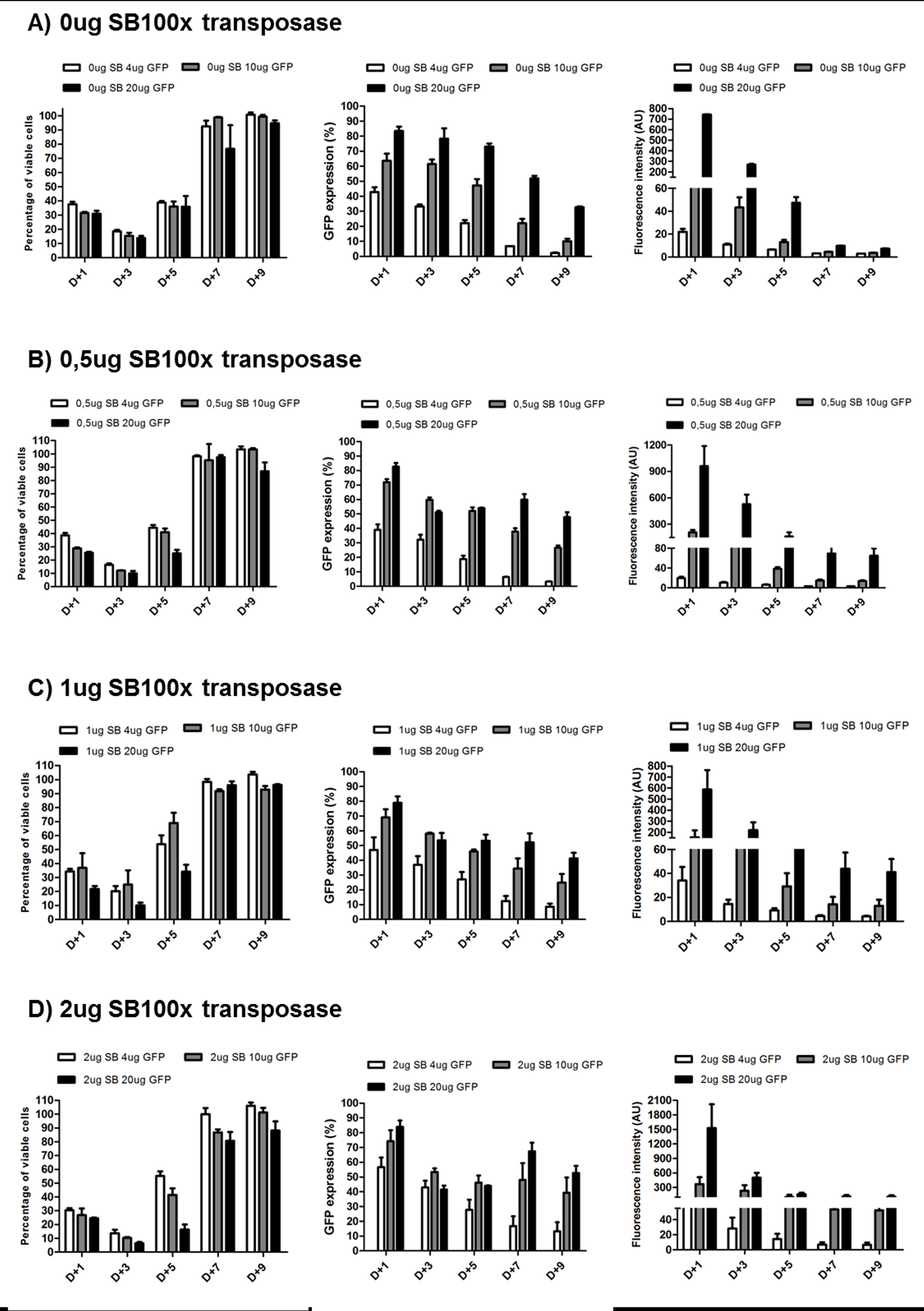

Supplement: Figure S3 — Impact of different transposon and transposase plasmid mass in viability and transgene expression. PBMCs from two healthy donors were electroporated using 1SM buffer, 4 µg, 10 µg or 20 µg of pT2-GFP plasmid and/or 0,5 µg, 1 µg or 2 µg of SB100x transposase plasmid. Cell viability and GFP expression were analyzed until d+9 by flow cytometry. Values are the average of two donors in triplicate and expressed as mean±SEM. Viability of electroporated cells were normalized to the negative control (not electroporated) cells. (TIF) [file pone.0060298.s003.tif]

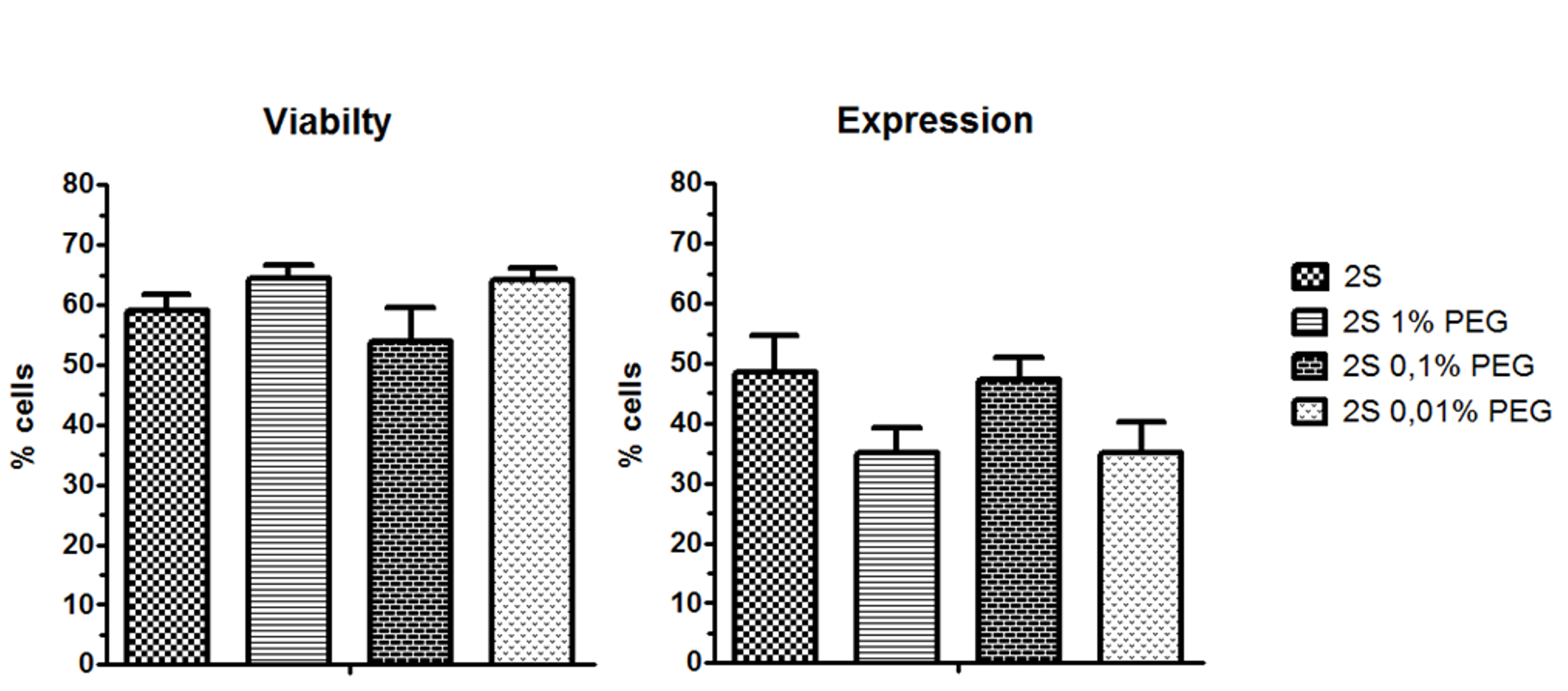

Supplement: Figure S4 — Electroporation of mouse lymphocytes in the presence of PEG. Total lymphocytes from lymph nodes of C57Bl/6 mice were isolated and electroporated using 2S buffers (supplemented or not with PEG) and 4 µg of pT2-GFP plasmid. Cell viability and GFP expression were analyzed after 2 h by flow cytometry. Data is representative of two independent experiments in triplicate. (TIF) [file pone.0060298.s004.tif]

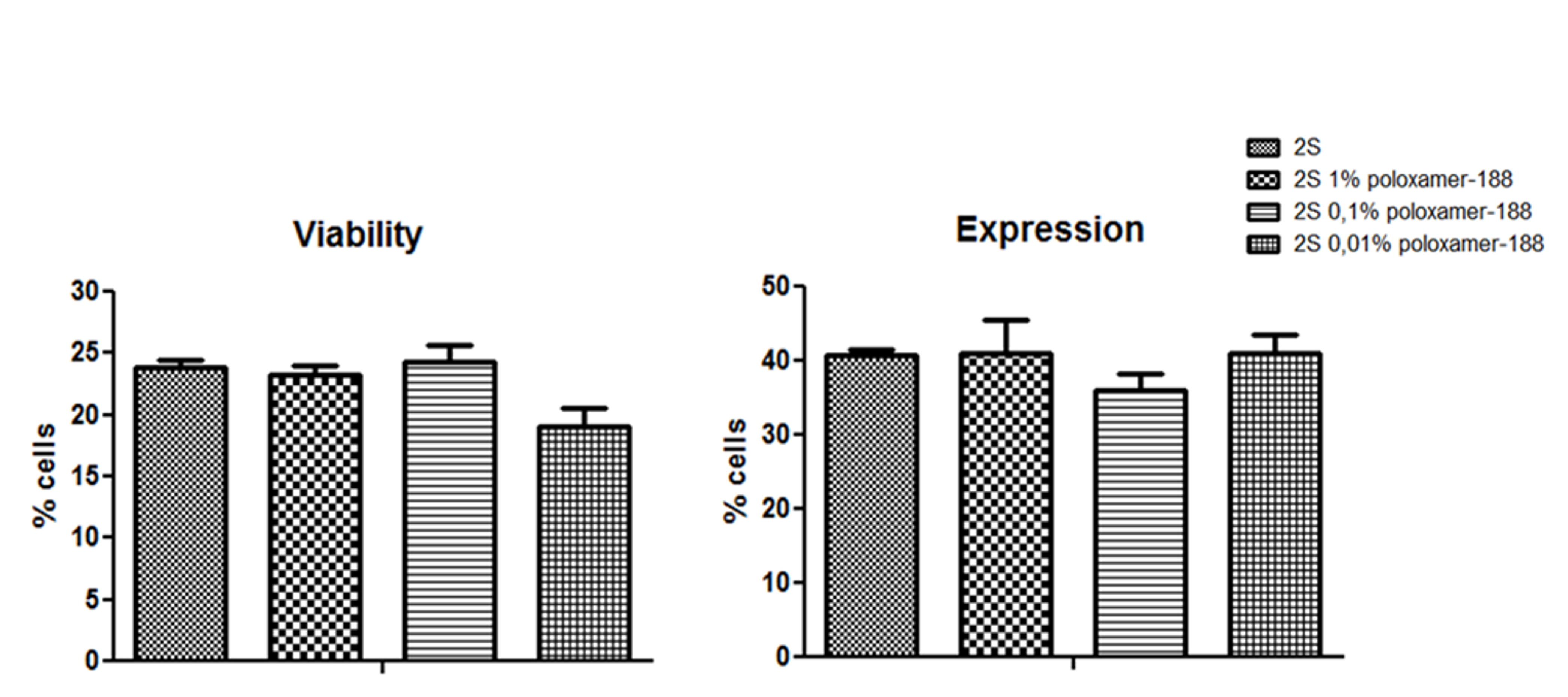

Supplement: Figure S5 — Electroporation of mouse lymphocytes in the presence of Poloxamer-188. Total lymphocytes from lymph nodes of C57Bl/6 mice were isolated and electroporated using 2S buffers (supplemented or not with Poloxamer-188) and 4 µg of pT2-GFP plasmid. Cell viability and GFP expression were analyzed after 24 h by flow cytometry. Data is representative of two independent experiments in triplicate. (TIF) [file pone.0060298.s005.tif]
