# Supplementary material for: An Efficient Low Cost Method for Gene Transfer to T Lymphocytes
Source: PLoS One. 2013 Mar 26;8(3):e60298. doi: 10.1371/journal.pone.0060298 (PMC3608570; doi:10.1371/journal.pone.0060298)
Supplement: Table S1 — Buffers used in electroporation experiments. (DOCX) [file pone.0060298.s006.docx]

**Table S1: Buffers used in electroporation experiments**

| **Buffer** | **Composition** |
| --- | --- |
| 1M | 5mM KCl; 15mM MgCl2; 120mM Na2HPO4/NaH2PO4 pH7.2; 50mM Manitol |
| 1S | 5mM KCl; 15mM MgCl2; 120mM Na2HPO4/NaH2PO4 pH7.2; 50mM Sodium Succinate |
| 1SM | 5mM KCl; 15mM MgCl2; 120mM Na2HPO4/NaH2PO4 pH7.2; 25mM Sodium Succinate; 25mM Manitol |
| 2M | 5mM KCl, 15mM MgCl2; 15mM HEPES; 150mM Na2HPO4/NaH2PO4 pH7.2; 50mM Manitol |
| 2S | 5mM KCl, 15mM MgCl2; 15mM HEPES; 150mM Na2HPO4/NaH2PO4 pH7.2; 50mM Sodium Succinate |
| 3P | 5mM KCl, 15mM MgCl2; 90mM NaCl; 10mM Glucose; 0,4mM Ca(NO3)2; 40mM Na2HPO4/NaH2PO4 pH 7,2 |
| 3H | 5mM KCl, 15mM MgCl2; 90mM NaCl; 10mM Glucose; 0,4mM Ca(NO3)2; 20mM HEPES and 75mM Tris/HCl |
