# Supplementary material for: An Efficient Low Cost Method for Gene Transfer to T Lymphocytes
Source: PLoS One. 2013 Mar 26;8(3):e60298. doi: 10.1371/journal.pone.0060298 (PMC3608570; doi:10.1371/journal.pone.0060298)
Supplement: Table S2 — Summary of the results obtained with in house buffers in different cell types. (DOC) [file pone.0060298.s007.doc]

Table S2: Summary of the results obtained with in house buffers in different cell types

| **Cell Type** | **Pre-activation** | **Buffer** | **Viability (D+1)** | **GFP expression (D+1)** | **Electroporation score** |
| --- | --- | --- | --- | --- | --- |
| Jurkat cell line | No | 3P | 18,3% | 67% | 61.8 |
| Primary human lymphocytes | No | 1SM | 67% | 43% | 57.6 |
| Primary murine lymphocytes | Yes | 2S | 47% | 39% | 92.3 |
